# Biomarkers Associated with Depression Improvement in Veterans with Gulf War Illness Using the Low-Glutamate Diet

**DOI:** 10.3390/nu16142255

**Published:** 2024-07-13

**Authors:** Amy A. Maury, Kathleen F. Holton

**Affiliations:** 1 Department of Neuroscience, American University, Washington, DC 20016, USA; am6140a@american.edu; 2 Department of Health Studies, American University, Washington, DC 20016, USA; 3 Center for Neuroscience and Behavior, American University, Washington, DC 20016, USA

**Keywords:** depression, dietary intervention, low-glutamate diet, Gulf War Illness, homocysteine, inflammatory cytokines, biomarkers

## Abstract

Gulf War Illness (GWI) is a chronic multi-symptom neurological disorder affecting veterans of the Gulf War that is commonly comorbid with depression. A secondary data analysis was conducted to examine serum homocysteine and inflammatory cytokines (IFN-γ, IL-6, IL-1β, TNF-α) as potential biomarkers of depression improvement among veterans with GWI after a one-month dietary intervention aimed at reducing excitotoxicity and increasing micronutrients. Analyses, including multiple linear and logistic regression, were conducted in R studio. Dietary adherence was estimated using a specialized excitotoxin food frequency questionnaire (FFQ), and depression was measured using the Center for Epidemiologic Studies Depression (CES-D) scale. After one month on the diet, 52% of participants reported a significant decrease in depression (*p* < 0.01). Greater dietary adherence (FFQ) was associated with increased likelihood of depression improvement; OR (95% CI) = 1.06 (1.01, 1.11), (*p* = 0.02). Reduced homocysteine was associated with depression improvement after adjusting for FFQ change (β = 2.58, *p* = 0.04), and serum folate and vitamin B12 were not mediators of this association. Reduction in IFN-γ was marginally associated with likelihood of depression improvement (OR (95% CI) = 1.11 (0.00, 1.42), (*p* = 0.06)), after adjustment for potential confounders. Findings suggest that homocysteine, and possibly IFN-γ, may serve as biomarkers for depression improvement in GWI. Larger trials are needed to replicate this work.

## 1. Introduction

Gulf War Illness (GWI) is a multi-symptom disorder characterized by widespread chronic pain, fatigue, headaches/migraines, cognitive dysfunction, and mood symptoms such as depression, anxiety, and post-traumatic stress disorder (PTSD). Of these, depression occurs in approximately one third of Gulf War veterans and is thought to contribute greatly to their decreased quality of life [1]. Deployed veterans of the Gulf War (GW) (1990–1991) are more than twice as likely as their non-deployed counterparts to experience depression, often comorbid with Gulf War Illness (GWI) [2,3]. Moreover, female veterans of the Gulf War have a significantly higher risk of suicide as compared to female non-Gulf War veterans and as compared with the general female U.S. population [4].

We have previously reported significant improvements in depression as a comorbid condition of GWI after one month on the low-glutamate diet (*p* < 0.001) [5]. The low-glutamate diet eliminates or significantly reduces foods containing excitotoxins, including free (unbound) glutamate and aspartate, while increasing whole foods that are high in nutrients known to be protective against excitotoxicity, including vitamins B2, B6, C, D, and E, omega-3 fatty acids, and the minerals magnesium and zinc. The glutamatergic system is thought to be dysregulated in depression [6]. Moreover, veterans of the Gulf War may have a higher susceptibility to dietary excitotoxins resulting from a compromised blood–brain barrier (BBB) as a result of neurotoxic exposures, head injuries, chronic stressors, and other insults related to military action [7]. Thus, a significant reduction in dietary excitotoxins may be able to reduce the chronic overactivation of the NMDA glutamate receptors thought to contribute to depression symptoms [8].

Previous studies have suggested that homocysteine and inflammatory cytokines may be potential biomarkers of interest for depression. Elevated homocysteine has been associated with depression in prior research [9,10,11,12]. It acts as a direct agonist of the NMDA-receptor, leading to dysregulated glutamatergic neurotransmission [9,13], and may worsen BBB permeability [12,14]. Further, insufficient levels of folate (B9) and/or vitamin B12 may result in elevated serum homocysteine via the methylation cycle. Similarly, depression is also associated with circulating inflammatory cytokines [15,16,17,18]. Recent studies have shown a causative relationship between inflammatory cytokines and the etiology of depression [19] and that those with inflammation-associated depression are more likely to experience treatment resistance [20].

The comprehensive dietary approach using the low-glutamate diet may be uniquely suited to improve dysregulated metabolic and immune pathways known to contribute to depression. Thus, the objective of this research was to understand whether homocysteine and/or the inflammatory cytokines IFN-γ, TNF-α, IL-6, and IL-1β, may be associated with depression improvement in veterans with GWI after one month on the low-glutamate diet.

## 2. Materials and Methods

### 2.1. Participants

This research used data from a prior completed randomized controlled clinical trial (NCT#03342482). A detailed consort flow diagram and recruitment information have been published previously [21]. The methods will be described here briefly. All participants were veterans of the U.S. Armed Forces, deployed to the Persian Gulf during the Gulf War (1990–1991), and were diagnosed with Gulf War Illness (GWI) based on both the Kansas case definition [22] and the CDC criteria [23]. Forty veterans with GWI were recruited from across the United States. To qualify, they had to be under 75 years old, have maintained stable medication usage for the last 3 months or more, have been actively deployed in the Gulf War, and be willing to change their diet while also discontinuing the use of alcohol, marijuana, and/or dietary supplements. Excluded subjects were those currently on active duty, active nicotine users, those who have been diagnosed with a seizure disorder (past or current) or substance use disorder (past year), those who took medication affecting glutamatergic or GABAergic neurotransmission, and/or those with severe asthma requiring hospitalization. All medications were held constant throughout the study.

### 2.2. Ethical Approval and Consent

The study from which these data were evaluated was conducted according to the guidelines laid down in the Declaration of Helsinki, and all procedures involving human subjects/patients were approved by the American University Institutional Review Board (IRB-2017-301) on 12 May 2017 and the Human Research Protection Office of the Department of Defense (DOD) (HRPO Log NumberA-20203.a) prior to recruitment of subjects. Written informed consent was obtained from all subjects. The study was registered at ClinicalTrials.gov as study ID no: NCT03342482.

### 2.3. Procedures

For the primary study, at baseline and post-diet, fasting blood samples and anthropometric measurements were obtained. Questionnaires were collected including the CES-D depression measure and a specially designed excitotoxin food frequency questionnaire (FFQ) (Table S1). Excitotoxin FFQ scoring is completed by summing up the self-reported excitotoxic intake during the reported period, with a higher score representing more frequent consumption of excitotoxic foods. Results are reported at baseline and again post-diet to estimate exposure to dietary free glutamate and aspartate intake and to provide an estimate of dietary adherence.

Upon completion of baseline assessments, participants were randomized into two groups: a dietary intervention group (which started the diet immediately) or a waitlisted control group (which started the diet one month later). Once participants were scheduled to start the diet, they received a detailed 2-h dietary training via Skype with the Principal Investigator, Dr. Kathleen Holton, the creator of the low-glutamate diet. A binder of information was also provided that included a detailed list of food additives to avoid, shopping ideas, recipes, and a list of foods that are highest in each micronutrient. An emphasis was placed on consuming foods high in vitamins B2, B6, C, D, and E, omega-3 fatty acids, and minerals magnesium, and zinc, since these nutrients are protective against excitotoxicity and the related outcomes of oxidative stress and inflammation. All measures were again completed post-diet.

As a measure of depression symptoms, participants completed the Center for Epidemiological Studies Depression (CES-D) self-report measure at baseline and post-diet. The CES-D was designed for screening purposes for epidemiological studies of the general population but is not used for clinical diagnoses [24]. The measure includes 20 questions that use a Likert-type scale that considers current or recent mood state, asking “How often this past week did you…”, and answers are rated 0 to 3. A score of (0) = rarely or none of the time (less than 1 day); (1) = some or a little of the time (1–2 days/week); (2) = occasionally/moderate amount of time (3–4 days/week); (3) = most or all of the time (5–7 days/week) [24]. Positive items are reverse-scored and answers are summed for a total score ranging from 0–60. A score greater than or equal to sixteen (16) is considered the threshold for depression.

Baseline and post-diet fasting blood samples were collected from participants using venous puncture. After being centrifuged, serum from the blood samples was extracted and aliquots were stored at −80 °C before shipping to the laboratories for analyses. Samples were tested for serum levels of homocysteine (μmoL/L), folate (ng/mL), vitamin B12 (pg/mL), and the cytokines IFN-γ, TNF-α, IL-6, and IL-1β (pg/mL). The cytokines were tested after thawing at INIM EM Papper Clinical Immunology Laboratory in Miami, FL using Quansys Biosciences custom 18-multiplex chemiluminescent assay (Quansys Biosciences, Logan, UT, USA) and plated in duplicate following the manufacturer protocols. Samples were tested for homocysteine at ZRT Laboratory in Beaverton, OR. The samples were plated in duplicate following protocols established by the manufacturer and, between plates, normalized using the internal controls run on each plate. Concentration calculations from standard curves that were created by a five-parameter weighted logistic regression were averaged for subsequent analyses.

### 2.4. Statistical Methods

While 40 participants participated in the primary study, this secondary analysis is based on the 33 participants (*n* = 33) who completed blood sample collection and the CES-D measures at their baseline and post-diet visits.

R Studio 2021.09.0 was used for all statistical analyses, and results were considered significant at *p* < 0.05. Corrections were not performed for multiple comparisons since the primary study was not powered specifically for these analyses. Linearity, independence, equality of error variance, and normality both visually and statistically were assessed for each biomarker variable.

Change scores were calculated for each biomarker variable by subtracting baseline concentrations from post-diet levels. The Shapiro–Wilk test of normality was used for each variable. For those that passed the test, baseline and post-diet paired *t*-tests were run, and Pearson correlations were evaluated for change in depression relative to change in each biomarker. For those variables that did not meet the test for normality, even after a power transformation, Spearman correlations were run. Multivariable linear regression was used to model the relation of change in each biomarker relative to change in depression score, after controlling for variables associated with depression, including age, sex, change in BMI, and dietary adherence measured by the excitotoxin FFQ.

Depression “improvement” was also assessed as a dichotomous variable, defined as either a reduction in the CES-D score by ≥30% or a post-diet CES-D score that changed from at or above the depression threshold of 16 [24,25] to below 16. Binary logistic regression was used to model the likelihood of depression improvement (odds ratios (OR) and 95% confidence interval (CI)) after adjustment for potential confounding factors, including age, sex, change in BMI, and dietary adherence measured by the excitotoxin FFQ. Biomarker variables were calculated using serum baseline levels minus post-diet levels for binary logistic regression.

Mediation analyses were conducted to explore whether folate and/or vitamin B12 would mediate the association between homocysteine and depression improvement since homocysteine production can be affected by dietary intake of folate and vitamin B12. These analyses were conducted using the R package “mediation” version 4.5.0 [26].

## 3. Results

### 3.1. Participant Demographics

Demographics of the 33 participants at the baseline visit are shown in Table 1. Study participants were predominantly Caucasian, with 67% of the sample being male, from four branches of the military, with an average age of 54 (6) years. At baseline, 76% of the study participants met the criteria for depression using the CES-D cut-off of 16 [24,25], with no differential observed based on sex (*p* = 0.67) or race (*p* = 0.68).

At baseline, there was no significant difference in depression scores between those taking antidepressant medication and those who were not taking medication (*p* = 0.30) and the mean CES-D scores for both groups were in the range that indicated depression (M = 32 (14) and M = 26 (14), respectively).

Biomarkers were compared at baseline to assess any differences based on depression symptom severity according to the CES-D cut-off (see Table 2). At baseline, serum homocysteine was the only biomarker to show a significant difference based on depression symptom score. Those who were depressed, based on the CES-D, had a median homocysteine level of 11 (5.7) µmol/L as compared to 9 (2.3) µmol/L for those not meeting the cut-off for depression (*p* = 0.03).

**Figure 1:**
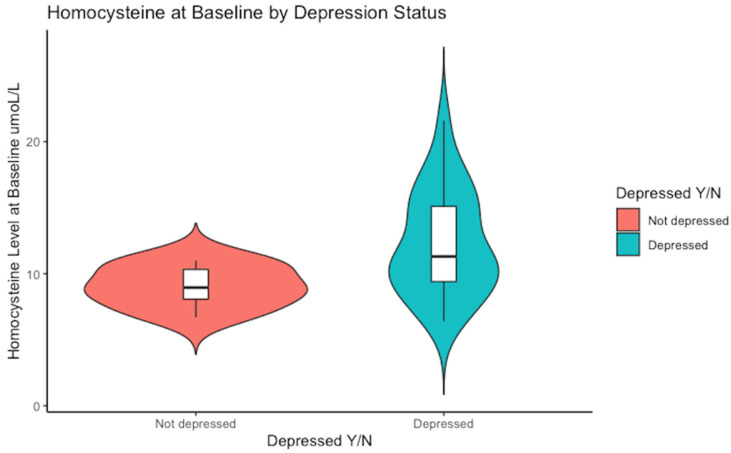
Baseline median concentration of serum homocysteine. The non-depressed group had a median value of Hcy of 9 µmol/L versus 11 µmol/L in the depressed sub-group, *p* = 0.03.

Notably, a higher range of serum homocysteine levels was evident in the subjects who met the cut-off score for depression, reaching as high as 22 µmol/L, whereas those who scored <16 on the CES-D scale peaked at 11 µmol/L (see Figure 1).

The baseline levels of the cytokines IFN-γ, TNF-α, IL-6, and IL1-β did not show significant differences when comparing groups with scores above versus below the threshold for depression. However, median serum IFN-γ was 13.9 (7) pg/mL in the depressed group versus 10.5 (4.3) pg/mL for those below the depression score threshold, a marginally significant difference (*p* = 0.10). An extreme outlier was observed for IFN-γ in one non-depressed subject who had a very high serum level that was 2.8 SDs above the mean. A sensitivity analysis was completed to evaluate the level of influence of this outlier on the overall results. After removal of the outlier, there was a significant difference in median baseline serum concentrations of IFN-γ in those who were depressed compared to those who were not depressed at baseline (13.9 pg/mL versus 9.9 pg/mL, respectively (*p* = 0.02)).

### 3.2. Change in Depression Score and Biomarkers after 1-month Dietary Intervention

Of the 25 participants who scored in the range for depression at baseline, 96% had a reduction in depression-symptom score by an average of 40% after one month on the low-glutamate diet. Participant dietary adherence was high based on the reported reduced intake of excitotoxic foods from baseline to post-diet, measured by the specialized excitotoxin FFQ score (*p* < 0.001). Table 3 shows the change in all measures from pre-diet to post-diet.

**Figure 2:**
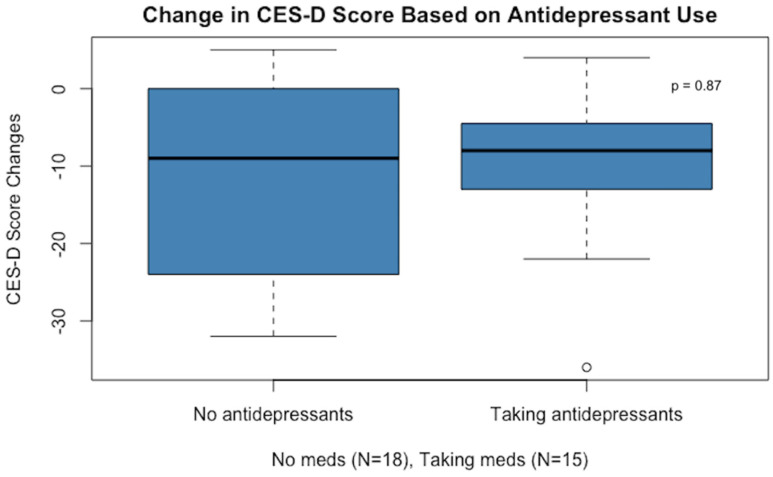
The change in mean depression scores for those subjects who did not take antidepressant medication (11-point mean drop on the CES-D scale) was similar to the change for those not taking medication (10-point drop). It should be noted that one participant’s score was an outlier (in the medication group), with a greater than 30-point reduction in their CES-D score.

After one month on the low-glutamate diet, participants had a significant reduction in depression scores as measured by the CES-D, and this was true whether or not participants were taking antidepressant medication (see Figure 2).

**Figure 3:**
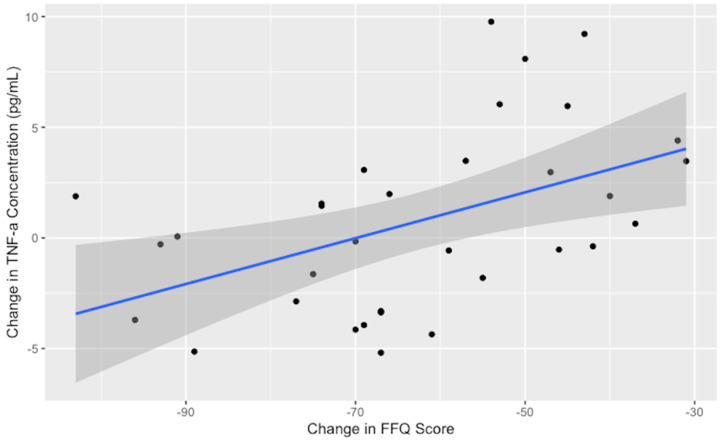
TNF-α and dietary adherence, measured as change in the excitotoxin FFQ (r = 0.50, *p* < 0.01). Greater reduction in free glutamate/aspartate in the diet was positively correlated with reduced serum TNF-α. The black dots represent individual participant excitotoxin FFQ score change (x-axis) and TNF-α blood concentration change (pg/mL) (y-axis). The blue line represents the smoothed trend line, with the 95% confidence interval shaded in gray.

No significant reductions were observed for homocysteine or the inflammatory cytokines after one month on the low-glutamate diet; however, IFN-γ was marginally reduced (*p* = 0.08). The cytokine TNF-α did not reach a level of significance in paired tests; however, the decrease in TNF-α concentrations was strongly correlated with excitotoxin FFQ dietary adherence (see Figure 3), indicating that as consumption of dietary excitotoxins is reduced, TNF-α levels are lowered.

The median serum folate concentration was significantly reduced from the dietary intervention (*p* = 0.04), but there was no evidence of dietary deficiency or insufficiency in folate either at baseline or post-diet. The reduction in folate was likely due to participants not adequately incorporating natural food sources of folate (e.g., green leafy vegetables, beans, etc.) as they reduced their intake of processed foods, which tend to be fortified with folic acid.

### 3.3. Binary Measure of Depression Improvement

**Figure 4:**
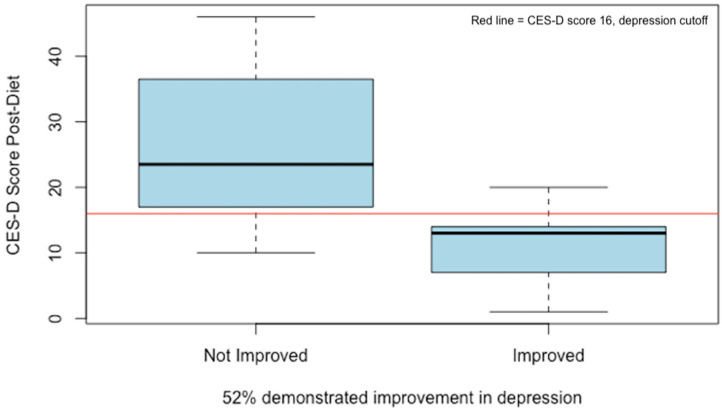
Post-diet CES-D scores based on a binary variable for depression improvement. A total of 52% of participants (*n* = 17) demonstrated ≥30% improvement in CES-D score and/or dropped below the threshold for depression (score of 16) indicated by the red line [27].

To better understand the biomarker changes that may have been associated with depression improvement, a binary measure was created based on either a reduction in CES-D score by 30% or a drop to below the cut-off of 16. By this measure, 52% of the study participants were considered “improved” (*p* = 0.02) after one month on the low-glutamate diet (see Figure 4).

Using the binary measure for depression improvement, Table 4 compares serum homocysteine, vitamin, and cytokine measures between those who did and did not experience improvement in depression scores after one month on the low-glutamate diet.

The reduction in dietary intake of excitotoxic foods was notably greater among those who had depression improvement, indicating a larger reduction in self-reported consumption of foods containing free glutamate and aspartate during the one-month intervention period (*p* < 0.01). Notably, serum homocysteine levels appeared to be reduced in those who had depression improvement, but increased in those who did not, though this did not reach statistical significance (*p* = 0.10). The cytokine IFN-γ also appeared to be more reduced in those who experienced depression symptom improvement compared to those who did not, but again, this biomarker change was shy of statistical significance (*p* = 0.08).

### 3.4. Modeling Results

A multivariable linear regression analysis showed that there was a significant association between change in total homocysteine and change in CES-D depression score after one month on the low-glutamate diet, after adjustment for potential confounding factors, (ß = 2.58, *p* = 0.04), F-statistic = 2.54 (5,27), *p* = 0.05, R^2^ = 0.32, with the proposed model:

CES-D Change = 17.8 + 2.58 * Hcy Change + 0.20 * FFQ Change + 4.97 * Sex − 0.32 * Age − 2.11 * BMI Change(1)

Folate and vitamin B12 were assessed for potential mediating effects on this linear relationship. While the direct effect of folate as a predictor had a marginally significant impact on homocysteine change (F = 3.345 (1, 31), (*p* = 0.08)), formal mediation was not supported since homocysteine change remained significant regardless of folate being included in the model. Furthermore, formal mediation by vitamin B12 was not observed since depression symptom improvement could not be attributed to changes associated with vitamin B12.

Logistic regression modeling was also used to assess the likelihood of depression improvement. The change in serum level of homocysteine was not associated with a significantly increased likelihood of depression improvement in logistic regression modeling: OR (95% CI) = 1.37 (−0.16, 0.79), (*p* = 0.20). However, a greater reduction in serum IFN-γ levels after one month on the low-glutamate diet was marginally associated with a higher likelihood of depression improvement: OR (95% CI) = 1.11 (0.00, 1.42), (*p* = 0.06) after controlling for age, sex, race, BMI change, and FFQ change.

## 4. Discussion

Biomarkers are needed to better understand and monitor the metabolic and immunoinflammatory changes that relate to depression symptom changes. This study showed that homocysteine and the inflammatory cytokine IFN-γ may serve as potential biomarkers for depression improvement among veterans with GWI.

There appeared to be strong adherence to the low-glutamate diet shown by the self-reported significant reduction in excitotoxic food intake. A greater reduction in excitotoxic food intake, as indicated by the excitotoxin FFQ change score, predicted greater depression improvement [28]. This is in alignment with animal research investigating the effect of glutamate on depression-like behavior. A rodent study demonstrated that exogenous glutamate delivered via food consumption, gavage, or intravenous administration resulted in behavioral changes and elevated symptoms of stress-induced depression behavior [6].

Homocysteine is directly related to glutamatergic dysregulation, where it has the ability to directly agonize the NMDA glutamate receptor [29], which can lead to depression symptoms [11,30]. Baseline serum homocysteine levels in our study reflected this relationship, with significantly higher levels of serum homocysteine in depressed subjects as compared to those without depression. Further, after one month on the low-glutamate diet, reduction in serum homocysteine was significantly associated with a reduction in the CES-D score.

Serum levels of homocysteine above 10 µmol/L have been associated with depression in several studies [13], including the Rotterdam Study involving 3,884 elderly subjects who were screened for depression using the CES-D [31]. In another study, median homocysteine levels of 14.7 µmol/L were found in 89 depressed adolescents and children, compared to 43 controls with levels of 10.2 μmol/L (*p* < 0.001) [32]. Moreover, higher serum homocysteine levels increase the odds of having depression, with the highest odds at levels above 15 mmol/L, according to the Hordaland Homocysteine Study involving 5948 adult subjects [33]. Elevated concentrations of homocysteine were also found to be a risk factor for depression in a two-year longitudinal study involving 732 Korean adults, wherein no interaction was evident between folate and vitamin B12 with homocysteine, and there was little evidence of confounding factors such as disability, cardiovascular disease, or lifestyle factors [34].

However, not all studies are consistent with these findings. In order to control for confounding factors related to homocysteine, a twin study involving U.S. veterans of the Vietnam War who were discordant for depression (*n* = 55 pairs) found no differences in homocysteine concentrations in the twin pairs based on depression status [30]. The researchers concluded that homocysteine levels were incidental to confounders, including high stress, reduced exercise, or other causes not directly associated with depression; however, a significant between-pair effect was observed, with higher depression scores (based on the Beck Depression Inventory (BDI)) being associated with higher homocysteine levels at the pair-level (*p* < 0.001) [30]. The Rotterdam Study also identified cardiovascular risk and functional disabilities as confounders for elevated homocysteine in depressed subjects [31].

Elevated levels of homocysteine are also associated with age, sex, race, high stress, and genetic abnormalities that cause homocystinuria; however, supplements of folate and SAM alone have been successfully used as therapeutic treatments for depression, suggesting that disrupted cellular methylation can be improved with dietary change and result in symptom improvement for certain depression sub-types [13].

Our study did not support the role of either folate or vitamin B12 as mediators for homocysteine change with depression improvement. No deficiency or insufficiency of either vitamin was evident at baseline or post-diet; therefore, the observed reduction in homocysteine post-diet should not be attributed to changes in serum folate or vitamin B12.

Serum levels of the cytokine IFN-γ were significantly reduced post-diet, and the levels of TNF-α also trended downward and strongly correlated with a greater reduction in consumption of excitotoxic foods, as measured by excitotoxin FFQ score change. Reduced cytokine levels may have influenced the activation of the kynurenine pathway. Dysregulation of the kynurenine pathway is a known factor contributing to the pathophysiology of depression. The inflammatory cytokines IFN-γ and TNF-α are suggested inducers for the kynurenine pathway that stimulate the expression of the enzyme indoleamine 2,3 di-oxygenase (IDO), which catabolizes tryptophan [35,36]. Activation of the kynurenine pathway shunts tryptophan away from the synthesis of serotonin and melatonin, and towards the production of kynurenine, which produces downstream metabolites, including neurotoxic quinolinic acid (QA), which can over-activate the NMDA glutamate receptor [37,38]. Evidence for the association between increased levels of the cytokine TNF-α and IDO has been found in rodent studies of stress-induced depression, where stress caused the upregulation of IDO in the cortex [36]. Upon the administration of an antidepressant, reduced symptoms of depression were accompanied by a reduction in both IDO and TNF-α levels in the frontal cortex [36]. Even stronger data suggest an association between IFN-γ and activation of IDO, with IFN-γ actually being used as an inducer of IDO in animal studies [35,39,40,41]. Moreover, there are strong data to suggest that IFN-γ treatment is an inducer of depression in cancer patients [42].

This is the first known study to evaluate changes in homocysteine and inflammatory cytokines relative to improvement in depression in veterans with Gulf War Illness. One of the strengths of this study was that females represented one third of participants in the study (33%). This is important since the Gulf War had a higher ratio of females than any prior military action and since female deployed veterans of the Gulf War have shown a higher risk of suicide than non-deployed Gulf War female veterans [4]. Participants also represented a wide range of geographical areas of the United States. There was evidence of adequate adherence to dietary protocols achieved by the participants as indicated by the change in the excitotoxin FFQ scores from baseline to post-diet (*p* < 0.001).

## 5. Limitations and Future Directions

This study was limited by its small sample size, which reduced statistical power for these analyses, and limited diversity of the sample. Future research should include a more ethnically and gender diverse sample. The balance of depressed versus non-depressed subjects was not equal due to this not being a criterion for entry into the study. Moreover, psychotherapeutic treatments were not assessed during the dietary intervention period, though participants were asked to keep their psychotherapy stable across the study period, similar to keeping their medication use stable. Also, many participants in this study had a BMI in the range of obesity (BMI > 30 kg/m^2^), which limits our ability to extrapolate these findings to normal-weight individuals. To analyze the potential confounding effect of obesity on the chronic release of inflammatory cytokines, future research should include participants not exhibiting obesity as a comorbid condition [43]. Future research could also include collection of the blood level of vitamins B6 and riboflavin (B2) to verify whether dietary nutrient composition may have improved the efficiency of the metabolic pathways of one-carbon metabolism. A measure of quinolinic acid produced by activation of the kynurenine pathway would also be pertinent to future research, as it may indicate whether inflammatory mechanisms are leading tryptophan metabolism away from the production of serotonin and melatonin, and toward the kynurenine pathway. A measure of plasma glutamate should also be included to verify whether dietary intake changes are correlated with a change in plasma levels of free glutamate, so that self-reported excitotoxin FFQ scores are not relied upon as the sole measure of dietary adherence.

## 6. Conclusions

This study demonstrated that serum homocysteine and the cytokine IFN-γ may serve as possible biomarkers for depression improvement in GWI using a low-glutamate diet. Both biomarkers were elevated at baseline in subjects with depression. Changes in serum homocysteine levels were significantly associated with CES-D score reduction, and the cytokine IFN-γ was marginally able to predict depression improvement after one month on the low-glutamate diet. Future larger-scale research is needed to better understand the metabolic and inflammatory mechanisms associated with depression in Gulf War veterans and to assess the viability and reliability of biomarkers as a tool to evaluate depression improvement. If results from future larger clinical trials confirm the use of the low-glutamate diet for depression improvement among veterans with Gulf War Illness, this diet could be used in the future as an adjunct clinical treatment for depression in this group. These results also suggest that future clinical trials to test this dietary approach as a treatment for depression alone may also be warranted.

## Figures and Tables

**Table 1:** Population description.

Demographic Variable (*n* = 33)	*n* (%)
Sex	
Female	11 (33%)
Male	22 (67%)
Race	
Caucasian	30 (91%)
Black/African American	3 (9%)
Military Branch	
Army	17 (52%)
Navy	6 (18%)
Air Force	5 (15%)
Marine Corp	5 (15%)
Depressed (CES-D)	
Yes	25 (76%)
No	8 (24%)
Antidepressant Medication *	
Yes	15 (45%)
No	18 (55%)
	Mean (SD)
Age (yrs.)	54.4 (5.7)
BMI (kg/mg^2^)	31.8 (5.7)
FFQ score	70.0 (18.8)
CES-D Score	28.6 (14.0)

* The following medications were reported by participants (all were held constant throughout the study): duloxetine, venlafaxine, sertraline, escitalopram, trazadone, and bupropion.

**Table 2:** Characteristics of subjects with depression at baseline.

Variable	Depressed * Yes *n* (%)	Depressed * No *n* (%)	*p*-Value **
	25 (76%)	8 (24%)	
Sex			0.67
Female	9 (82%)	2 (18%)	
Male	16 (73%)	6 (27%)	
Race			0.68
Caucasian (91%)	23 (76%)	7 (23%)	
Black/African American (9%)	2 (67%)	1(33%)	
	Median (IQR)		*p*-value **
Age (years)	53 (6)	57.5 (6)	0.34
BMI at baseline (kg/mg^2^)	31.0 (8.7)	31.2 (5.1)	0.58
FFQ at baseline	76 (19)	56 (27)	0.64
Homocysteine (μmoL/L)	11.3 (5.7)	9.0 (2.3)	**0.03**
Folate (ng/mL)	13.5 (9.9)	16.3 (7.6)	0.34
Vitamin B12 (pg/mL)	559 (301)	763 (461)	0.40
Cytokines:			
TNF-α (pg/mL)	2.6 (4.9)	1.8 (1.7)	0.30
IFN-γ (pg/mL)	13.9 (7.0)	10.5 (4.3)	*0.10*
IL-6 (pg/mL)	2.4 (1.8)	3.1 (1.9)	0.27
IL-1β (pg/mL)	11.2 (4.3)	11.0 (3.1)	0.92

* “Depressed” at baseline is defined as a score of ≥16 using CES-D. ** Mann–Whitney. Bold font indicates significant difference. Italics indicates marginally significant differences based on alpha 0.05.

**Table 3:** Changes from baseline to post-diet (*n* = 33).

	Pre-Diet (Baseline)	Post-Diet	*p*-Value *
	Mean (SD)		
CES-D Score	28.6 (14.1)	18.5 (12)	**<0.001**
Dietary Adherence (Excitotoxin FFQ)	70.0 (18.8)	7.3 (9.0)	**<0.001**
Homocysteine (μmoL/L)	11.5 (3.7)	11.5 (3.4)	0.85
Folate (ng/mL)	14.7 (6.3)	13.0 (5.4)	**0.04**
TNF-α (pg/mL)	3.1 (2.7)	3.9 (3.3)	0.30
IFN-γ (pg/mL)	13.7 (5.6)	11.4 (4.3)	*0.08*
	Median (IQR)		*p*-value ^†^
Vitamin B12 (pg/mL)	572 (333)	574 (445)	0.72
IL-6 (pg/mL)	2.9 (1.5)	2.6 (0.9)	1.00
IL1-β (pg/mL)	11.2 (4.3)	9.9 (3.2)	0.78

* Paired samples *t*-test. ^†^ Wilcoxon signed-rank test. Bold font indicates significant difference; italic text indicates marginally significant differences based on alpha of 0.05.

**Table 4:** Characteristics of depression improvement.

Variable	Depression Improved ^1^		
	Yes	No	
	17 (52%)	16 (48%)	
	Median (IQR)		*p*-Value ^†^
Age (years)	54 (8.0)	53 (7.5)	0.34
BMI Change (kg/mg^2^)	−0.47 (0.85)	−0.41 (1.0)	0.94
Excitotoxin FFQ Change	−70.0 (10)	−54.5 (18)	**<0.01**
Homocysteine Change (μmoL/L)	−0.60 (1.5)	0.55 (1.3)	*0.10*
Folate Change (ng/mL)	−0.70 (4.6)	−4.4 (6.4)	*0.07*
Vitamin B12 Change (ng/mL)	−1.0 (217)	−15.5 (140)	0.80
Cytokine Change:			
TNF-α (pg/mL)	−0.29 (4.9)	1.9 (4.6)	0.20
IFN-γ (pg/mL)	−5.0 (11.8)	0.31 (8.3)	*0.08*
IL-6 (pg/mL)	0.01 (1.5)	0.20 (2.1)	0.82
IL1-β (pg/mL)	0.67 (6.2)	−0.39 (3.4)	0.89

^1^ Depression improvement is defined as a ≥30% reduction in CES-D scores and/or a drop from at or above the threshold for depression (score of 16) to below the threshold. ^†^ Wilcoxon rank-sum test. Bold font indicates significant difference; italic font indicates marginally significant difference based on alpha of 0.05.

## Data Availability

This study was registered at https://clinicaltrials.gov/study/NCT03342482 (accessed on 12 January 2021).

## Supplementary Materials

The following supporting information can be downloaded at https://www.mdpi.com/article/10.3390/nu16142255/s1, Table S1: Excitotoxin Food Frequency Questionnaire.
