# Supplementary material for: Biomarkers Associated with Depression Improvement in Veterans with Gulf War Illness Using the Low-Glutamate Diet
Source: Nutrients. 2024 Jul 13;16(14):2255. doi: 10.3390/nu16142255 (PMC11280460; doi:10.3390/nu16142255)
Supplement: Supplementary file 1 [file nutrients-16-02255-s001.zip › nutrients-3078311-supplementary.pdf]

Supplementary Materials

Table S1. Excitotoxin Food Frequency Questionnaire

ID # ____________________ Date ________________

Food Frequency Questionnaire

Please indicate how frequently you consumed each food item by marking the appropriate box.

	1x/mo	2x/mo	1x/wk	2x/wk	3-5x/wk	Daily	≥2/day
Protein powder, protein shakes, or any drink with added protein							
Spice mixes or mixed seasoning packets (like taco seasoning mix)							
Marinades purchased at grocery store (like teriyaki sauce, BBQ sauce etc.)							
Boxed foods including seasoning packets (like mac n/cheese, Rice a Roni, Hamburger Helper, etc.)							
Canned goods (canned soup, chili etc.) – do not include plain beans or plain vegetables							
Broth or bouillon (chicken/beef/bone or vegetable broth, bouillon cubes, etc.)							
Frozen prepared foods (not including plain frozen vegetables or fruit)							
Spaghetti/pizza sauce or tomato sauce							
Asian food (sushi, Chinese, Thai, etc.)							
Soy sauce, or soy sauce alternatives like Bragg’s Amino Acids/Coconut Aminos							
Other Asian sauces (like oyster sauce, fish sauce, etc.)							
Worcestershire Sauce							
Seasoned nuts/seeds (other than just salted, like tamari almonds, BBQ sunflower seeds, etc.)							
Chips (except for plain tortilla or plain potato chips)							
Crackers (other than plain saltines or plain Triscuits)							
Store-bought salad dressing or salad dressing mixes other than Caesar							
Caesar salad dressing (even homemade)							
Croutons or other seasoned salad topper							
Anchovies or sardines							
Parmesan, Romano, sharp cheddar cheese, or other aged cheeses							
Processed meat (sausage, pepperoni, bacon, ham, hot dogs, deli meat, jerky)							

Office Use Only: Score = _________________

ID # ____________________ Date _________________

Equal (aspartame sweetener)							
Diet Soda							
Chewing gum							
Breath mints							
Vitamins/medication in gelatin capsules							
Chewable vitamins/medications							
Sugar-free candy							
Jello or Jello pudding							
Gelatin Candy (gummy worms, skittles, marshmallows, etc.)							
Flavored juice drink or vegetable juice (includes V8, Crystal Light, and any flavoring added to water) – does not include flavored seltzer water like La Croix or Bubbly							
Reduced calorie "Light" flavored yogurt							
Regular flavored yogurt (like Dannon/Yoplait/Activia)							

Office Use Only: Score = __________________
